# Hereditary kidney tumor syndromes: structured evaluation of a questionnaire-based approach

**DOI:** 10.1093/ckj/sfag143

**Published:** 2026-05-06

**Authors:** Jan Degenhardt, Theresa von Zehmen, Bodo Beck, Florian Erger, Axel Heidenreich, Roman-Ulrich Müller, Pia Paffenholz

**Affiliations:** Department II of Internal Medicine, University of Cologne, Faculty of Medicine and University Hospital Cologne, Cologne, Germany; Research Center On Rare Kidney Disease (RECORD), University Hospital Erlangen, Erlangen, Germany; Department of Urology, Uro-Oncology, Robot-Assisted and Reconstructive Urologic Surgery, University of Cologne, Faculty of Medicine and University Hospital Cologne, Cologne, Germany; Institute of Human Genetics, University Hospital Cologne and University of Cologne, Faculty of Medicine, Cologne, Germany; Institute of Human Genetics, University Hospital Cologne and University of Cologne, Faculty of Medicine, Cologne, Germany; University Medicine Greifswald, Institute for Molecular Genomics, Core Unit Genomics, Greifswald, Germany; Department of Urology, Uro-Oncology, Robot-Assisted and Reconstructive Urologic Surgery, University of Cologne, Faculty of Medicine and University Hospital Cologne, Cologne, Germany; Department of Urology, Medical University Vienna, Austria; Department II of Internal Medicine, University of Cologne, Faculty of Medicine and University Hospital Cologne, Cologne, Germany; Cluster of Excellence on Cellular Stress Responses in Aging-associated Diseases (CECAD), University of Cologne, Cologne, Germany; Department of Nephrology, Medical Faculty, University Hospital Düsseldorf, Heinrich-Heine-University Düsseldorf, Düsseldorf, Germany; Department of Urology, Uro-Oncology, Robot-Assisted and Reconstructive Urologic Surgery, University of Cologne, Faculty of Medicine and University Hospital Cologne, Cologne, Germany; Center for Integrated Oncology (CIO) Köln-Bonn, Cologne, Germany

**Keywords:** familial renal cancer, genetic risk assessment, genetic screening, hereditary renal cell carcinoma, tumor predisposition syndrome

## Abstract

**Background:**

Up to 8% of renal tumors have a monogenic cause, yet hereditary renal cell carcinoma (hRCC) syndromes such as von Hippel–Lindau (VHL), Tuberous Sclerosis Complex (TSC), Birt–Hogg–Dubé (BHD), and Hereditary Leiomyomatosis and Renal Cell Cancer remain underdiagnosed. Early diagnosis is critical for patient management, genetic counseling, and family screening. We developed and prospectively validated a structured risk assessment tool (hRCC score) for identifying patients at risk of hereditary renal tumors.

**Methods:**

A prospective single-center study was conducted at the University Hospital Cologne (2020–2022) including 200 patients with histologically confirmed renal tumors. The hRCC score incorporated age at diagnosis, multifocal/bilateral disease, histology, extrarenal manifestations, and family history. Patients with a score ≥1.5 were referred for genetic testing using a multiplex MLPA (Multiplex Ligand-dependent Probe Amplification)-based panel including *TSC, MET, VHL, FH, SDH-A-D*, and *FLCN*.

**Results:**

Of 195 eligible patients, 34.4% (*n* = 67) had a high-risk hRCC score (≥1.5). Overall, 71 (36.4%) underwent genetic testing; a pathogenic or likely pathogenic variant was detected in 50.7% of tested patients, corresponding to 18.5% of the total cohort. The most common diagnoses were TSC (58.3%), VHL (16.7%), and BHD (11.1%). Confirmed hereditary cases had significantly higher mean hRCC scores (4.67 vs 0.48, *P* < .0001). Extrarenal manifestations and bilateral or multifocal disease were the strongest predictors. The cutoff of 1.5 yielded 97.2% sensitivity and 79.8% specificity.

**Conclusions:**

The hRCC score is an effective clinical screening tool for detecting patients at risk for hereditary renal tumors, demonstrating high diagnostic yield and supporting targeted referral for genetic evaluation.

KEY LEARNING POINTS **What was known:**

- Generally, 5%–8% of renal tumors have a monogenic cause, but hereditary renal cell carcinoma (hRCC) cases (e.g. von Hippel–Lindau, Birt–Hogg–Dubé, and Hereditary Leiomyomatosis and Renal Cell Cancer) are frequently underdiagnosed.
- Early diagnosis changes management and enables family screening and/or counseling.
- Genetic testing depends on clinical suspicion or expert referral. No standardized screening tools are widely adopted to guide referrals for genetic testing.

**This study adds:**

- Prospective single-center evaluation of a questionnaire-based hRCC screening tool. With a referral cutoff of ≥1.5, the screening tool achieved 97.2% sensitivity and 79.8% specificity.
- Out of the 71 patients referred for testing, a genetic cause was confirmed in 36 representing 18.5% of the total cohort.
- Strongest predictors of a hereditary kidney cancer syndrome were extrarenal manifestations and bilateral/multifocal lesions.

**Potential impact:**

- Our hRCC score tool provides a simple and effective screening workflow to standardize referrals for genetic testing.
- It will enable earlier diagnosis and syndrome-specific management for affected individuals and families.
- This approach may focus resources on high-yield referrals for genetic testing.

## INTRODUCTION

Renal cell cancer (RCC) is the sixth most common form of cancer [1, 2] and consists of histopathological and molecular subtypes [3]. Sporadic RCC manifests at a median age of 64 years and occurs more frequently in men. Smoking, obesity, and hypertension, but also chronic kidney disease, especially requiring hemodialysis, predispose to the development of kidney cancer [1, 2, 4]. However, RCC can also occur as part of hereditary cancer syndromes: 3%–5% of RCC cases are suspected to occur due to pathogenic variants in cancer susceptibility genes [5]. Hereditary kidney cancer syndromes such as von Hippel–Lindau disease (VHL), Tuberous Sclerosis Complex (TSC) , Birt–Hogg–Dubé syndrome (BHD), or Hereditary Leiomyomatosis and Renal Cell Cancer (HLRCC) have a high penetrance and expressivity of extrarenal manifestations while others, such as Hereditary Papillary Renal Cancer (HPRC), seem to be limited to a renal phenotype [6, 7]. Hereditary kidney cancer generally manifests at an earlier age and more often in advanced stages with bilateral, multifocal, or recurring disease [6]. Hereditary renal tumors show different clinical features: hereditary renal cell carcinoma (hRCC) can be highly aggressive with rapid growth and early metastasis, e.g. in HLRCC and SDH (Succinate dehydrogenase) -deficient RCC. Other tumors, such as the hybrid-oncocytic or chromophobe renal tumors observed in BHD or the clear-cell RCC in VHL show slower growth and tend to metastasize later. Angiomyolipomas (AMLs) associated with TSC are overwhelmingly benign but are still associated with major bleeding and may cause loss of renal function or life-threatening complications. Screening and treatment strategies are tailored to specific hRCC syndromes and range from active surveillance of lesions smaller than 3 cm to early margin resections of suspicious lesions [8]. Establishing the correct diagnosis is therefore critical in guiding management of these patients. Yet, even among known monogenic disorders, underdiagnosis as well as a long patient journey until receiving the diagnosis are common. This comes with major shortcomings in patient and family counseling. Consequently, improving diagnostic performance is an important goal toward optimal patient care, but remains a challenge considering constraints in time and education on these specific diseases. Currently, no comprehensive assessment guiding genetic testing is routinely employed. The need to improve diagnostic completeness was recognized by the German Cancer Society, resulting in the creation of a structured evaluation form. This form has entered the national certification process of cancer centers and is referred to in the national guidelines [30].

This risk assessment tool is designed to allow nonexpert personnel regarding hRCC to identify patients at high risk (HR) for hereditary kidney tumor syndromes and to provide guidance for follow-up specialist referral and care and genetic testing. Here, we present a single-center experience with the implementation of this score-based structured risk assessment tool.

## MATERIALS AND METHODS

### Development of the risk assessment tool

The risk assessment tool was developed with the objective to identify individuals at risk for hRCC or associated syndromes. We calculated a score (hRCC score) weighing personal cancer history including age at diagnosis, presence of multifocal/bilateral tumors, RCC histology, and extrarenal manifestations linked to hereditary kidney tumor syndromes. Family history was assessed regarding the presence of kidney tumors in first- or second-degree relatives and known hereditary kidney tumor syndromes. For each item, 1 point was awarded except age at diagnosis and known hereditary kidney tumor syndrome counting 1.5 points.

### Patient recruitment

We performed a prospective single-center cohort study including patients presenting to the Department of Urology or the Department of Nephrology for evaluation or surgery of localized kidney tumors from 2020 to 2022. Patients were screened for the risk of hereditary kidney cancer syndromes using the questionnaire-based tool. Only patients with tumor histology were included in the study (Table 1; see Supplementary Table 1 for excluded cases).

**Table 1:** Baseline characteristics.

		Total	hRCC score ≥1.5	hRCC score <1.5	Sig.
		195	67 (34.4%)	128 (65.6%)	
Age (SD)		58.8 (14.9)	45.4 (11.7)	65.9 (11.1)	**
Sex	Female	77 (%)	35 (17.9%)	42 (21.5%)	*
	Male	118 (%)	32 (16.4%)	86 (44.1%)	
Renal tumor type	ccRCC	104 (53.3%)	28 (14.4%)	76 (39.0%)	*
	pRCC	29 (14.9%)	5 (2.6%)	24 (12.3%)	*
	chRCC	11 (5.6%)	4 (2.1%)	7 (3.6%)	*
	AML	28 (14.4%)	22 (11.3%)	6 (3.1%)	*
	Oncocytoma	12 (6.2%)	3 (1.5%)	9 (4.6%)	*
	Other	11 (5.6%)	5 (2.6%)	6 (3.1%)	*
T stage	T1a	61 (31.3%)	2 (10.8%)	40 (20.5%)	ns
	T1b	39 (20.5%)	7 (3.6%)	33 (16.9%)	ns
	T2a	11 (5.6%)	2 (1.0%)	9 (4.6%)	ns
	T2b	4 (2.0%)	1 (0.5%)	3 (1.5%)	NA
	T3a	23 (11.8%)	6 (3.1%)	17 (8.7%)	ns
	T3b	5 (2.6%)	1 (0.5%)	4 (2.1%)	NA
	T4	3 (1.5%)	0	3 (1.5%)	NA
	Tx	48 (24.6%)	29 (14.9%)	19 (9.7%)	ns
N stage	N0	2 (1.0%)	2 (1.0%)	0	NA
	N1	12 (6.2%)	0	12 (6.2%)	NA
	Nx	171 (92.8%)	65 (33.4%)	116 (59.5%)	*
M stage	M0	51 (26.1%)	10 (5.1%)	41 (21.0%)	ns
	M1	30 (15.4%)	9 (4.6%)	21 (10.8%)	ns
	Mx	114 (58.5%)	48 (24.6%)	66 (33.8%)	ns
Grading (ISUP)	1	57 (29.3%)	12 (6.2%)	45 (23.1%)	ns
	2	50 (25.6%)	11 (5.6%)	39 (20.0%)	ns
	3	16 (8.3%)	4 (2.1%)	12 (6.2%)	ns
	4	2 (1.0%)	0	2 (1.0%)	NA
	N/A	60 (30.8%)	36 (18.5%)	24 (12.3%)	ns
	Missing	10 (5.2%)	4 (2.1%)	6 (3.1%)	ns
Bilateral		48 (24.6%)	34 (17.4%)	14 (7.2%)	*
Multifocal		61 (31.2%)	35 (17.9%)	26 (13.3%)	*
Relapse		57 (29.3%)	37 (19.0%)	20 (10.3%)	*

Percent values related to total sample size. Significance between hRCC score groups reported as * *P* < .05; ** *P* < .01; or ns as for nonsignificant comparisons. ccRCC: clear-cell renal cell carcinoma, chRCC: chromophobe renal cell carcinoma, “other”: see Supplementary Table 1 for category breakdown.

Patients with an hRCC score of ≥1.5 were referred for genetic testing (HR). Patients with an hRCC score of 1 were clinically reevaluated for syndromes associated with hereditary kidney cancer and referred to genetic testing based on expert clinical evaluation. Patients with no risk factors for hereditary kidney cancer syndromes received standard follow-up. All analyses performed were approved by the ethics committee of the Medical Faculty (University of Cologne, identifier: 23-1465-retro).

### Genetic testing

Patients identified as at risk for hereditary kidney tumor syndromes received pretest counseling and informed consent was obtained before genetic testing took place. Genetic testing was conducted using a multiplex ligand-dependent probe amplification-based panel of *TSC, MET, VHL, FH, SDH-A, SDH-B, SDH-C, SDH-D*, and *FLCN*. ClinVar and Human Gene Mutation Database were used for classification of variants.

A genetic diagnosis was considered as confirmed upon detection of a class 4 (likely pathogenic) and/or class 5 (pathogenic) variant. For TSC, additionally the clinical assessment as “definite TSC” according to the 2021 Diagnostic Criteria [20] was deemed confirmatory, even in the absence of an American College of Medical Genetics and Genomics (ACMG) class 4 or 5 variant.

Patients without detection of a P/LP variant after referral for genetic testing were assessed as “no genetic finding,” including patients who declined genetic testing or were lost to follow-up. “No mutation detected” was used to describe the absence of P/LP variants after genetic testing.

Secondary findings were reported according to the ACMG recommendations [29]. After establishing a diagnosis, a multidisciplinary follow-up was initiated in coordination with the relevant medical specialties.

### Statistical analysis

We presented continuous variables as median (25th–75th percentile) and categorical variables as *n* (%). All reported *P*-values are two-sided, and *P*-values <.05 were considered statistically significant. Significance was assessed using Student’s *t*-test or chi-squared tests where applicable. Odds ratios were calculated using Firth logistic regression. Statistical analysis was performed using the R language and environment with the added libraries ggplot2, networkD3, dplyr, tidyr, patchwork, viridis, ComplexUpset, ggsignif, logistf, gt, stringr, and broom. All packages are available from the CRAN repository.

## RESULTS

**Figure 1:**
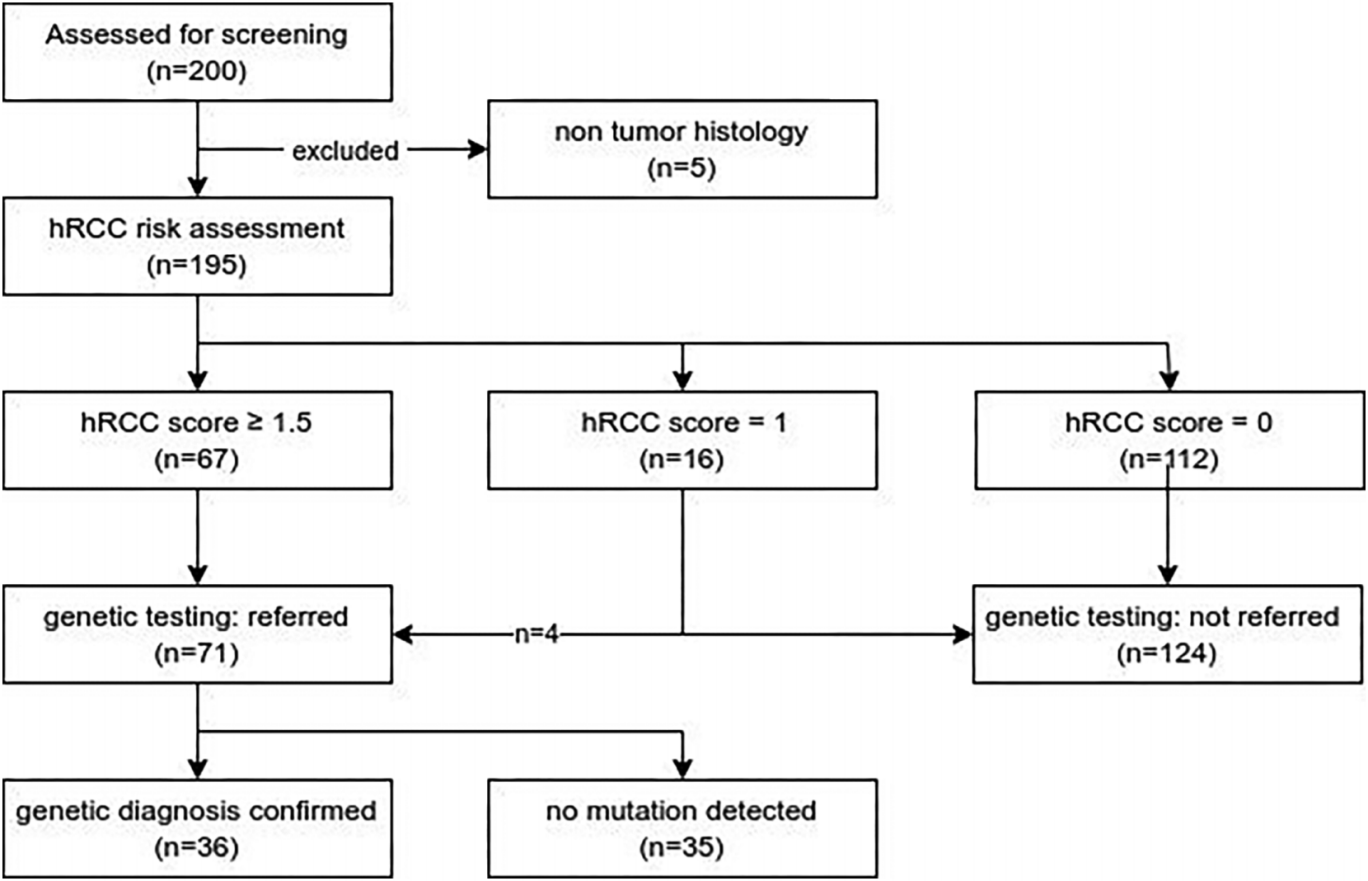
Consort diagram for hRCC screening and genetic testing referral. A total of 200 individuals were initially screened. Five individuals were excluded due to nontumor histology, leaving 195 patients eligible for hRCC risk assessment. Seventy-one patients were referred for genetic testing resulting in confirmation of a hereditary kidney tumor syndrome in 36 patients.

A total of 200 patients were screened for risk assessment of which five were excluded due to nontumor histology (Supplementary Table 1). Of 195 patients who underwent hRCC risk assessment, 67 patients (34.4%) had an hRCC score of 1.5 or more (HR) and consequently received the advice to obtain genetic testing. Patients with an hRCC score of <1.5 were deemed low risk (LR). In the LR group, 16 patients with an hRCC score of 1 were referred for reevaluation by an expert clinician and 4 of these were then further referred for genetic testing (Fig. 1).

The mean age of the cohort was 58.8 (±14.9) years, with patients in the HR group being significantly younger (45.4 years ± 11.7) compared to the LR group (65.9 years ± 11.1). The overall sample showed an uneven gender distribution with 77 patients (39.5%) being female, and 118 patients (60.5%) being male. The gender distribution in the HR subgroup was more balanced with 35 females to 32 male patients. The individuals in the HR group had a higher percentage of bilateral (17.4% vs 7.2%) and multifocal (31.2 vs 17.9%) tumors. Relapsing RCC was also more frequent in the HR group (29.3% vs 10.3%) compared to the LR group (Table 1).

**Figure 2:**
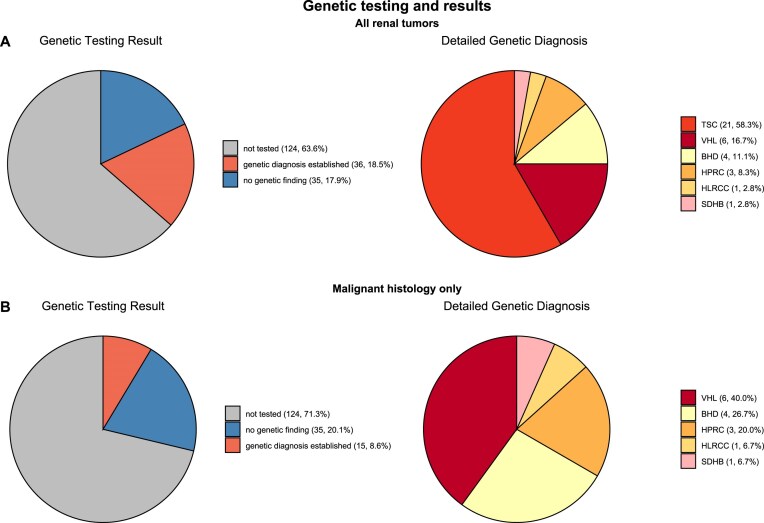
Genetic testing and results. (A) Out of the total cohort, 71 patients (36.4%) were referred for genetic testing. Among those tested, 36 patients received a confirmed genetic diagnosis. The most frequent diagnosis was TSC. (B) After exclusion of benign kidney tumors (exclusively AMLs in the sample), 50 patients (28.7%) were referred for genetic testing with a confirmed genetic diagnosis in 15 patients. SDHB: SDH-B deficient renal cell carcinoma.

A disease-causing variant was detected in 36 patients (18.5%) of the total cohort, representing 50.7% of 71 patients (36.4%) referred for genetic testing. Sixteen patients (22.5%) did not complete genetic testing following referral, owing to either declined genetic counseling or loss to follow-up (Supplementary Fig. 1). The most common hereditary kidney tumor syndromes were TSC (21 patients, 58.3% of tested patients), followed by VHL disease (six patients, 16.7%) and BHD syndrome (four patients, 11.1%). More rare kidney tumor syndromes were HLRCC (2.8%, one patient), HPRC (8.3%, three patients), and SDH-B (2.8%, one patient) (Fig. 2A). Details on clinical characteristics, TNM (tumor-node-metastasis) stages, and histomorphologic subtypes in cases with a confirmed genetic diagnosis are depicted in Supplementary Fig. 2.

A subgroup analysis including only malignant renal cell carcinomas saw a referral rate of 29.1% with 31.1% of genetic assessments resulting in a confirmed diagnosis (Fig. 2B).

Patients with a confirmed genetic diagnosis had a significantly higher hRCC risk score (mean 4.67) than the rest of the cohort (mean 0.484, *P* < .0001).

**Figure 3:**
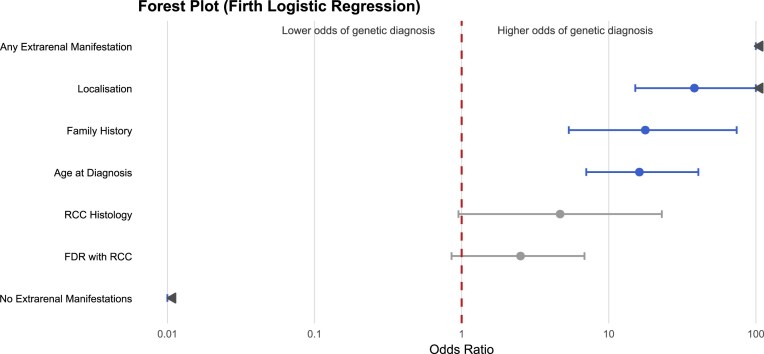
Predictors of genetic diagnosis in hRCC patients. Significant predictors of a genetic diagnosis were presence of any extrarenal manifestation, localization, family history, and age at diagnosis. First-degree relatives with RCC and RCC histology showed a nonsignificant tendency toward presence of a hereditary kidney tumor syndrome. FDR: first-degree relative.

Extrarenal manifestations were strongly associated with the confirmation of a hereditary kidney tumor syndrome. Similarly, criteria related to tumor frequency and localization, such as bilateral or multifocal RCC and relapsing disease, as well as family history and age at diagnosis, were also significant indicators. Histopathological features of RCC and having a first-degree relative with RCC were also suggestive of a hereditary kidney tumor syndrome, though these associations did not reach statistical significance (Fig. 3  and  Supplementary Table 2].

**Figure 4:**
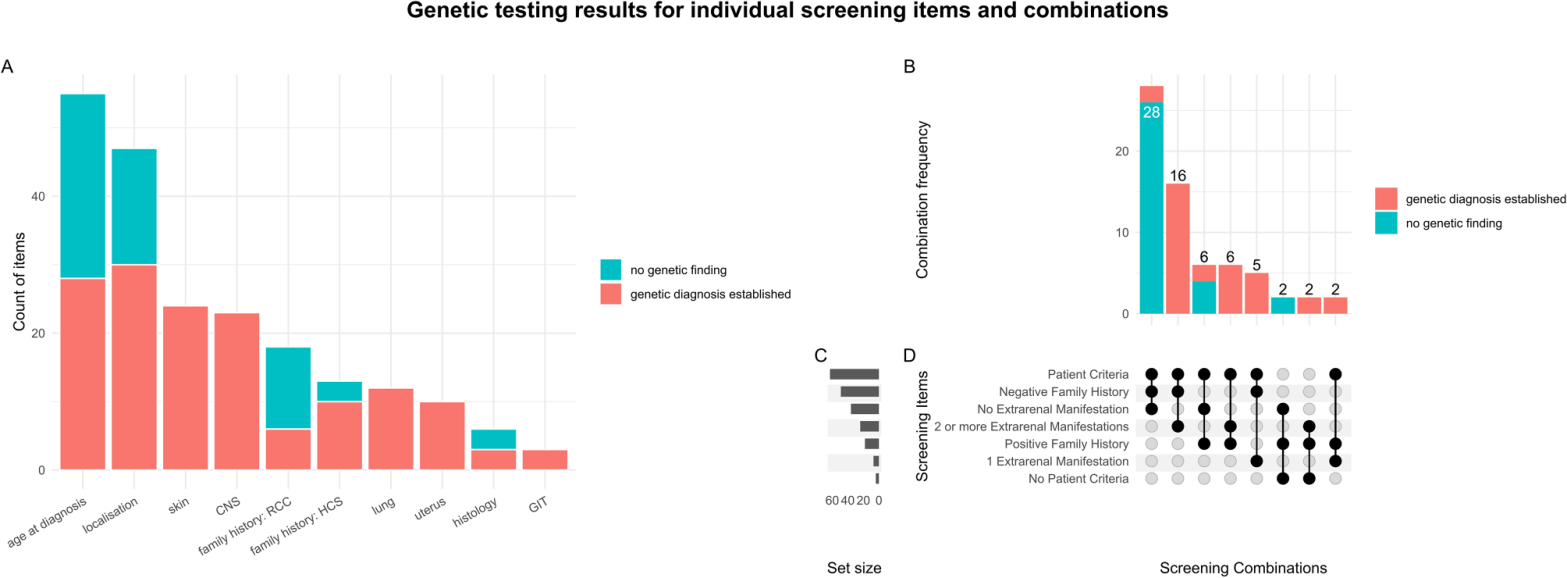
Genetic testing results for individual screening items and combinations. (A) Counts of positive individual screening items and genetic testing results for each count. The highest yield of genetic testing was observed for extrarenal manifestations reported in the hRCC screening tool. (B) Upset plot indicating the combinations of screening items and genetic testing results. Patient criteria were age at diagnosis, tumor localization, and histopathologic features. Family history included known hereditary kidney tumor syndromes and history of RCC in first-degree relatives. All 28 patients testing negative fulfilling only patient criteria were referred to genetic testing due to young age at diagnosis only (<46 years). (C) Frequency of criteria. (D) Matrix indicating item combinations. HCS: hereditary cancer syndrome, GIT: gastrointestinal tract.

For all individuals with skin (*n* = 24), central nervous system (CNS; *n* = 22), lung (*n* = 12), uterine (*n* = 10), and gastrointestinal (*n* = 3) manifestations, genetic diagnostics confirmed a disease-causing variant (Fig. 4A). Of all individuals with a positive family history of hereditary kidney tumor syndromes (*n* = 13), 76.9% (10/13) had a confirmed genetic diagnosis, the remaining three patients were lost to follow up before genetic testing. A family history of RCC (*n* = 12) was associated with a diagnosis of hereditary kidney tumor in 33.3% (6/18) of cases. Histopathology features associated with hRCC were present in six cases, with a genetic diagnosis established in 50% (3/6) (Fig. 4A  and  Supplementary Table 3) with one case of BHD, SDH-B, and HLRCC, respectively.

An analysis of the combinations of hRCC screening items and the outcomes of genetic testing in referred patients revealed differences based on whether the risk indicators were patient-related criteria (age at diagnosis, RCC histology, and localization), family-related criteria (first-degree relative with RCC or a confirmed hRCC syndrome), or extrarenal manifestations (Fig. 4B). The absence of both family-related criteria and extrarenal manifestations resulted in a confirmed diagnosis in only two cases. Among the cases, in which a diagnosis could not be established, the common factor was the absence of extrarenal manifestations.

The predefined cutoff value for referral to genetic diagnostics of 1.5 provided a sensitivity of 97.2% and specificity of 79.8% (Table 2).

**Table 2:** Sensitivity and specificity of the hRCC risk assessment tool at various cutoff values.

Cutoff	Sensitivity	Specificity
1.0	1	0.704
1.5	0.972	0.798
2.0	0.972	0.911
2.5	0.972	0.924
3.0	0.916	0.993
3.5	0.888	0.993

**Figure 5:**
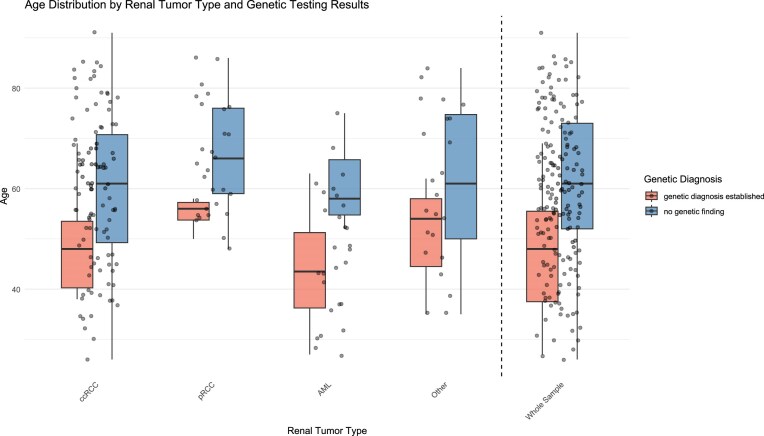
Age distribution by renal tumor type and genetic testing results. Patients with a confirmed genetic diagnosis tend to have a younger age at diagnosis across pRCC, ccRCC, and AML. For other tumor entities no significant association was found, likely due to low number of cases for rare kidney tumor entities. ccRCC: clear-cell renal cell carcinoma.

Patients with hRCC were generally younger across histology types. However, this difference only reached statistical significance in patients with papillary renal cell carcinoma (pRCC) and AML (Fig. 5 and Supplementary Table 4].

## DISCUSSION

Identification of hRCC is clinically relevant, as management strategies differ from sporadic RCC and among specific hRCC syndromes. Clinical consequences for patients include nephron-sparing approaches, partial nephrectomy with either margin resection or enucleation or nephrectomy but also affect the timing of interventions, surveillance, and further screening [17–21]. Additionally, specific systemic treatments for specific forms of hRCC become available [9, 10]. While hRCC forms mostly share manifestation at an earlier age and family histories associated with the autosomal dominant patterns of inheritance [11], other features, such as extrarenal manifestations or distinct pathologic features show either varying penetrance and expressivity or histomorphologic variability [12]. Any single one or a combination of these risk factors can warrant a referral for genetic risk assessment and testing for hRCC syndromes [13]. However, structured assessment of these risk profiles is difficult outside expert settings and often not implemented in clinical routine. This problem was recognized by the German Cancer Society leading to the setup of a structured evaluation form allowing for such an assessment in standard nonexpert care.

Our sample closely resembles the established demographics of RCC [1, 2, 4]. In accordance with the autosomal dominant hereditary nature of hRCC-related conditions, the individuals in our HR group were younger and had a more balanced gender distribution.

In this prospective single-center study, we identified 34.4% of the cohort as patients with an increased risk for hRCC syndromes using our hRCC risk assessment tool. Another study by Kushnir *et al*. [14] found 35% of patients at risk for hRCC-related conditions utilizing a prospective database. In our study, 36 (50.9%) individuals representing 18.5% of the whole cohort were confirmed to have a hereditary condition underlying the RCC. The higher percentage of confirmed hRCC diagnosis to previous literature reports is likely owed to the gap in referrals for genetic testing that Kushnir *et al*. identified in their database-driven design. Besides, our center is a tertiary care provider, i.e. may be enriched for complex cases. Nonetheless, this finding highlights the importance of follow-up referral and genetic testing in individuals at risk for hRCC.

In our risk assessment, we observed extrarenal manifestations being highly predictive for presence of a hereditary kidney tumor syndrome while age, being the most common risk factor in the sample, showing only 50.9% positive predictive value. Nonetheless, we would still advocate for the inclusion of age in any risk assessment for hRCC due to the general availability as opposed to extrarenal manifestations, which can be missed if not assessed by an experienced physician or if they are not present in the individual or specific hRCC syndrome, e.g. HPRC.

For breast cancer, assessment for hereditary predisposition is the clinical standard: in this regard, several guidelines recommend BRCA1/BRCA2 testing if criteria similar to our risk assessment tool are met [26]. Depending on the specific guideline, a likelihood of 5%–10% of a pathogenic or likely pathogenic BRCA1/BRCA2 variant should motivate genetic testing [27, 28].

Considering estimates from literature of 5%–8% of RCC being hereditary, similar recommendations for RCC might be justified. Our reported rate of malignant hereditary kidney tumors of 8.6% corresponds with these estimates and exceeds literature estimates for all renal tumors (18.5%) [25]. Negative health impacts of hereditary kidney cancer syndromes are not limited to cancer associated morbidity as is demonstrated, e.g. by CNS or retinal lesions in VHL and TSC. Since extrarenal manifestations mostly precede renal neoplasms in these conditions, timely diagnosis is critical for affected families. We demonstrated a high diagnostic yield for TSC, VHL, and BHD, but did also find rare syndromes (HLRCC, HPRC, and SDH-D).

Limitations of our study are the single-center design, resulting potential referral bias, and limited ethnic diversity. Therefore, further studies are needed to validate the screening tool in larger, multicenter cohorts. Nonetheless, it is likely that structured assessment of these risk factors will help guiding diagnostic (and eventually also therapeutic) decision-making in various settings.

Another research question of critical interest to assist in further development is to identify the false-negative, e.g. patients not meeting hRCC criteria but harboring confirmed P/LP variants in hRCC susceptibility genes. To warrant wide-spread genetic testing, longitudinal studies to assess the impact of early genetic diagnosis on patient outcomes, including survival and quality of life, are essential. Besides, current false-positive cases, i.e. with a score >1.5 but without genetic findings, may indeed be resolved by genome-sequencing approaches in the future and should be revisited periodically, e.g. every 5 years.

hRCC risk assessment will need to adapt as more clinical data on hRCC syndromes [22, 23] are available and new hRCC-related genes are discovered [24]. Alongside hRCC, gene panels will need updating or switching to exome sequencing entirely. We believe that our data show a high diagnostic yield for a standardized risk assessment for hRCC with the promise to support routine care in filling the current diagnostic gap.

## Supplementary Material

sfag143_Supplemental_Files

## Data Availability

The data underlying this article will be shared on reasonable request to the corresponding author.
