# Supplementary material for: Hereditary kidney tumor syndromes: structured evaluation of a questionnaire-based approach
Source: Clin Kidney J. 2026 May 6;19(6):sfag143. doi: 10.1093/ckj/sfag143 (PMC13254475; doi:10.1093/ckj/sfag143)
Supplement: sfag143_Supplemental_Files [file sfag143_supplemental_files.zip › HereditaryKidneyCancerScreeningQuestionnaire.pdf]

Supplement: Hereditary kidney cancer risk assessment questionnaire

This questionnaire is intended to aid in assessing the risk of a hereditary kidney cancer syndrome in patients presenting with kidney tumors.

Histology:

renal cell carcinoma (specify)

○ clear cell ○ papillary ○ chromophobe ○ other ○ unknown

○ oncocytoma ○ hybrid (specify):

○ angiomyolipoma: ○ other (specify):

Results/recommendation:

>= 1,5 points: refer to a center experienced in managing hereditary kidney cancer or initiate genetic workup

>= 1 point: consider referral to a center experienced in managing hereditary kidney cancer or genetic workup

Consult other specialties for evaluation of extrarenal findings as needed. (e.g., dermatology, pathology, pulmonology).

**Personal medical history**			
Affected organs	Finding/localization	check if applicable	score
Kidney tumor *age at diagnosis*	<= 46 years		1,5
Kidney tumor *localization*	Bilateral tumor[s]		1
	Multifocal tumor (>=3 lesions) [a]		1
	Bilateral and multifocal tumors [a]		1
Kidney tumor *histology*	non-clear cell RCC and specific histologic findings [b]		1
Skin/mucosa	any: leiomyoma, fibrofolliculoma, trichodiscoma,<br>shagreen patch, periungual fibroma, oral fibroma, angiofibroma [c]		1
Adrenal glands	Pheochromocytoma or paraganglioma		1
Lung	Lymphangioleiomyomatosis or cysts or recurrent pneumothorax		1
CNS	CNS or retinal hemangioblastoma, pediatric tumor-associated epilepsy		1
GI	Gastrointestinal stromal tumor		1
Uterus	Multiple leiomyoma or fibroid tumors (onset <= 30 years)		1
**Family history**			
Kidney tumors in 1st or 2nd degree relatives [d]			1
Known hereditary kidney cancer syndrome [e]			1,5
Total score			

[a] except papillary RCC in end-stage renal disease (usually bilateral/multilocular occurrence without inheritability)

[b] relevant histopathologic findings in non-clear cell RCC are:
- ➔ hybrid oncocytic RCC (oncocytoma and chromophobe RCC)
- ➔ HLRCC associated RCC (fumarate hydratase deficiency)
- ➔ SDHB-deficient RCC

[c] Naevi, melanoma, basal cell carcinoma and squamous cell carcinoma do not qualify. If unclear, consult dermatology prior to genetic testing.

[d] First degree relatives: parents, siblings, children
Second degree relatives: grandparents, grandchildren, aunts/uncles, nieces/nephews, half-siblings

[e] confirmed diagnosis of e.g.:
Von-Hippel-Lindau syndrome (VHL)
Birt-Hogg-Dubé syndrome (BHD)
Hereditary leiomyomatosis and renal cell carcinoma (HLRCC)
Hereditary papillary renal cell carcinoma (HPRCC)
Tuberous sclerosis complex (TSC)
Hereditary paraganglioma/pheochromocytoma
Cowden syndrome / PTEN-hamartoma tumor syndrome
Urothelial carcinoma in hereditary nonpolyposis colorectal carcinoma
