# Supplementary material for: Hereditary kidney tumor syndromes: structured evaluation of a questionnaire-based approach
Source: Clin Kidney J. 2026 May 6;19(6):sfag143. doi: 10.1093/ckj/sfag143 (PMC13254475; doi:10.1093/ckj/sfag143)
Supplement: sfag143_Supplemental_Files [file sfag143_supplemental_files.zip › hRCC Screening_v7 - Supplements.pdf]

Overcoming the diagnostic gap for hereditary kidney tumor syndromes: Structured evaluation of a questionnaire-based approach

Jan Degenhardt[1]

Theresa von Zehmen[2]

Bodo Beck[3]

Florian Erger[3]

Axel Heidenreich[2,#]

Roman-Ulrich Müller[1,4,#,*]

Pia Paffenholz[2,*]

[1] Department II of Internal Medicine, University of Cologne, Faculty of Medicine and University Hospital Cologne, Cologne, Germany.

[2] Department of Urology, Uro-Oncology, Robot-Assisted and Specialized Urologic Surgery, University Hospital Cologne, Cologne, Germany.

[3] Institute of Human Genetics, University Hospital Cologne and University of Cologne, Faculty of Medicine, Cologne, Germany

[4] Cluster of Excellence on Cellular Stress Responses in Aging-associated Diseases (CECAD), University of Cologne, Cologne, Germany.

# Corresponding authors

* Shared last authorship

Supplements

Supplemental Table 1: specification of “other” renal tumors and excluded cases (see Table 1)

Renal tumor type: “other”	count
cystic RCC	2
high-grade sarcoma	1
melanoma metastasis	1
hybrid oncocytic tumor	5
metanephric adenoma	1
Medullary cystic RCC	1

Renal tumor type: excluded cases	count
benign cyst	3
cholesterol granuloma	1
accessory spleen	1

RCC: renal cell carcinoma

Supplemental Table 2: Odds ratios (OR) and 95% confidence intervals (CI) for variables associated with a confirmed genetic diagnosis in hereditary renal cell carcinoma (hRCC) patients. (Supplement to Fig. 3)

Variable	CI low	CI high	p-value
No Extrarenal Manifestations	3.232552e-06	3.902296e-03	0.000000e+00
Any Extrarenal Manifestation	2.562594e+02	3.093531e+05	0.000000e+00
Family History	5.348397e+00	7.405046e+01	1.462550e-06
FDR with RCC	8.539657e-01	6.843546e+00	9.123869e-02
Age at Diagnosis	7.037641e+00	4.065432e+01	3.337886e-12
Localisation	1.515129e+01	1.102130e+02	0.000000e+00
RCC Histology	9.523384e-01	2.294894e+01	5.689738e-02

FDR: first-degree relative, RCC: renal cell carcinoma, CI: confidence interval, OR: Odds ratio

Supplemental Table 3: Individual hRCC score screening items and rate of established diagnosis (Supplement to Fig. 4A)

Screening item	Total	Diagnosis established	No mutation detected
age at diagnosis	55	28 (50.9%)	27 (49.1%)
localisation	38	30 (79%)	8 (21%)
skin	24	24	0
CNS	22	22	0
family history: HCS	13	10 (76.9%)	3 (23.1%)
family history: RCC	12	6 (60%)	6 (50%)
lung	12	12	0
uterus	10	10	0
histology	6	3 (50%)	3 (50%)
GIT	3	3	0

CNS: central nervous system, RCC: renal cell carcinoma, HCS: hereditary cancer syndrome. GIT: gastrointestinal tract.

Supplemental Table 4: Age distribution by Renal Tumor Type and Genetic Testing Result. (Supplement to Fig. 5)

Renal tumor type	Age: diagnosis confirmed	Age : no mutation detected	Age: all patients	p-value (corrected)	p-value
ccRCC	49.3 (11.7)	60.0 (14.9)	59.3 (14.9)	0.385	0.0770
pRCC	55 (3.6)	67.0 (10.8)	65.3 (10.9)	0.0036	0.000720
chRCC	67	61.9 (16.5)	62.4 (15.7)	NA	NA
AML	44.1 (11.2)	60.8 (8.9)	47.7 (12.7)	0.0164	0.00329
other	50.3 (13.9)	61.6 (15.3)	60.1 (15.4)	1	0.292
total	47.3 (11.5)	61.4 (14.4)	58.8 (14.9)	0.0000000315588	0.0000000315

ccRCC: clear cell renal cell carcinoma, pRCC: papillary renal cell carcinoma, chRCC: chromophobe renal cell carcinoma, AML: Angiomyolipoma

Supplemental Figure 1: Consort diagram of patients referred for genetic testing

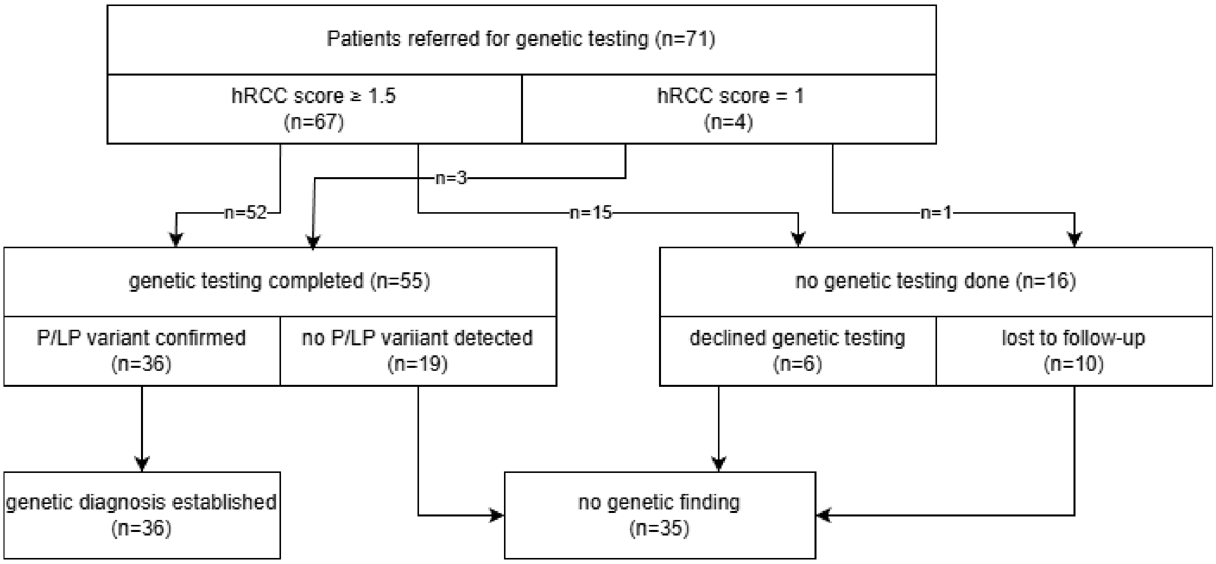

Supplemental Figure 1: Consort diagram detailing pathways to genetic diagnosis or no genetic finding. Sixteen out of 71 patients (22,5%) referred for genetic testing did not complete it.

Supplemental Figure 2:

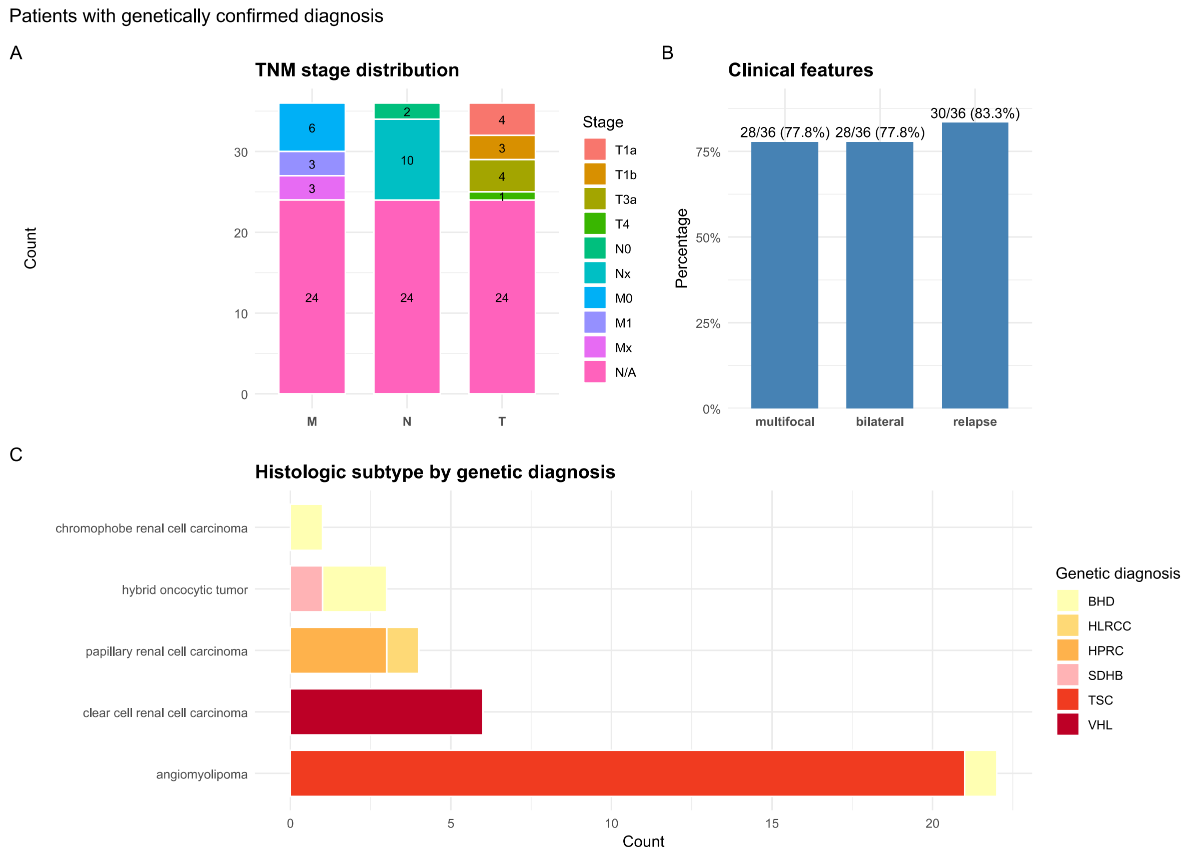

Supplemental Figure 2: Clinical and (histo-)morphological findings in patients with genetically confirmed diagnosis. A: Distribution of TNM stages B: Presence of multifocal, bilateral or relapsing renal tumors. C: Histologic subtypes and respective diagnosis. Histologic subtypes largely matched the expected spectrum in their respective syndromes, e.g. clear-cell renal cell carcinoma for VHL or angiomyolipoma for TSC. BHD: Birt-Hogg-Dubé, HLRCC: Hereditary Leiomyomatosis with Renal Cell Carcinoma, HPRC: Hereditary Papillary Renal Cancer, SDHB: SDH-B-deficient Renal Cell Carcinoma, TSC: Tuberous Sclerosis Complex, VHL: Von-Hippel-Lindau.
